# Templating synthesis of Fe_2_O_3_ hollow spheres modified with Ag nanoparticles as superior anode for lithium ion batteries

**DOI:** 10.1038/s41598-017-08773-6

**Published:** 2017-08-29

**Authors:** Xiaoping Lin, Jianmin Zhang, Xiaobin Tong, Han Li, Xi Pan, Peigong Ning, Qiuhong Li

**Affiliations:** Pen-Tung Sah Institute of Micro-Nano Science and Technology, Xiamen University, Xiamen, 361005 China

## Abstract

Ag-Fe_2_O_3_ hollow spheres are synthesized by using Ag@C core-shell matrix as sacrificial templates. The morphologies and structures of the as-prepared samples are characterized by scanning electron microscopy, X-ray powder diffraction energy dispersive, transmission electron microscopy and high resolution transmission electron microscopy. In contrast to Fe_2_O_3_ hollow spheres, Ag-Fe_2_O_3_ hollow spheres exhibit much higher electrochemical performances. The Ag-Fe_2_O_3_ composites exhibit an initial discharge capacity of 1030.9 mA h g^−1^ and retain a high capacity of 953.2 mA h g^−1^ at a current density of 100 mA g^−1^ after 200 cycles. Furthermore, Ag-Fe_2_O_3_ electrode can maintain a stable capacity of 678 mA h g^−1^ at 1 A g^−1^ after 250 cycles. Rate performance of Ag-Fe_2_O_3_ electrode exhibits a high capacity of 650.8 mA h g^−1^ even at 5 A g^−1^. These excellent performances can be attributed to the decoration of Ag particles which will enhance conductivity and accelerate electrochemical reaction kinetics. Moreover, the hollow structure and the constructing particles with nanosize will benefit to accommodate huge volume change and stabilize the structure.

## Introduction

In recent decades, a lot of studies had been triggered in developing high-performance electrode materials with high energy density, high power density, long lifetime and low cost^1–4^. Since the first report by Tarascon *et al*.^5^, transition metal oxides (TMOs) had been identified as promising candidates for lithium ion batteries due to their low conversion potential, high specific capacity and environmental friendliness^6–10^. Among the TMOs, Fe_2_O_3_ was considered as a promising anode for lithium ion batteries (LIBs) because of its low cost, high theoretical capacity (1007 mA h g^−1^), environmental protection and nontoxicity^11–13^. As evidenced by countless research works, the high capacity was mainly obtained by the reversible conversion reaction between Fe_2_O_3_ and Li^+^^14^. Recently, there had been a great deal of progress in the study of Fe_2_O_3_ based electrode material^15–18^. Lou *et al*. fabricated carbon-coated α- Fe_2_O_3_ hollow nanohorns on the CNT backbone, which greatly improved the electrochemical properties of Fe_2_O_3_ electrode^16^. However, the commercial applications of Fe_2_O_3_ in LIBs were impeded by the sluggish conversion reactions, large volume expansion and contraction during the charge and discharge cycles^19^. Two strategies had been developed to overcome these significant drawbacks. One was to retain a large deal of void space by synthesizing porous/nanostructured anode materials (e.g. nanotubes^20, 21^, mesoporous materials^22, 23^, nanopeapods^24^), which presented to accommodate the volume change and shorten the Li^+^ transport distance. Thus, they could exhibit enhanced rate capability and improved cycle retention^25^. Another was to coat native materials with carbon^26^ or decorate some conductive materials to increase the electrical conductivity and alleviate aggregation^27–29^. Kim *et al*. fabricated Ag-Li_4_Ti_5_O_12_ nanofibers by electrospinning, which displayed enhanced rate capability and cycling stability compared to the bare Li_4_Ti_5_O_12_^28^. To the best of our knowledge, a hollow structure of Fe_2_O_3_ nanospheres decorated with Ag nanoparticles had never been reported, and we expected the introduction of Ag could improve the electrochemical properties of Fe_2_O_3_.

In recent years, template-based method was widely used in preparing hollow nanostructured materials^30–32^. In this work, we successfully synthesized Ag-Fe_2_O_3_ hollow nanospheres by using Ag@C core-shell matrix as sacrificial templates. The removal of carbon layer and surface diffusion during the annealing process was the main reason for the formation of hollow nanostructures. As the temperature up to 600 °C, Ag core started to melted and partial hollow core formed^33^. Ag-Fe_2_O_3_ composites displayed a unique hollow structure which obviously shortened the Li^+^ transport distance and effectively slowed down the volume expansion. The incorporation of Ag could effectively improve the conductivity of the material. Compared with Fe_2_O_3_ hollow nanospheres, Ag-Fe_2_O_3_ hollow nanospheres displayed enhanced cycling properties and excellent performance at high rates.

## Results and Discussions

**Figure 1:**
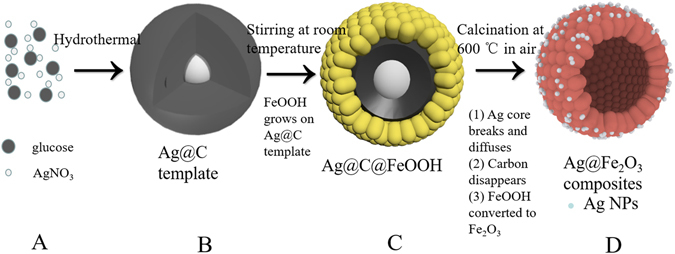
Schematic illustration of a tentative mechanism for the template-directed synthesis of the Ag decorated Fe_2_O_3_ nanospheres.

The schematic illustration of a tentative mechanism for the template-directed synthesis of the Ag modified Fe_2_O_3_ nanospheres is shown in Fig. 1. Initially, we synthesize Ag@C templates by hydrothermal method (A → B). Then, Ag@C templates are coated with FeOOH by stirring at room temperature (B → C). In the course of stirring, CH_3_COO^−^ hydrolysis to produce OH^−^, then, Fe^2+^ react with OH^−^ and O_2_, and ultimately form FeOOH (${{\rm{CH}}}_{3}{{\rm{COO}}}^{-}+{{\rm{H}}}_{2}{\rm{O}}\to {{\rm{CH}}}_{3}{\rm{COOH}}+{{\rm{OH}}}^{-},4{{\rm{Fe}}}^{2+}+8{{\rm{OH}}}^{-}+{{\rm{O}}}_{2}\to 4{\rm{FeOOH}}+2{{\rm{H}}}_{2}{\rm{O}}$).Next, during the calcinations at air (C → D), with the disappearance of carbon layer, Ag core breaks and diffuses, in the meantime, FeOOH converts to Fe_2_O_3_. Eventually, Ag-Fe_2_O_3_ hollow spheres are fabricated. The formation of Fe_2_O_3_ hollow structure decorated with Ag nanoparticles can be related with the removal of carbon template and surface diffusion processes. During the annealing, along with the disappearance of the carbon layer, Ag core begin to melt and shrink, leading to the presence of lots of Ag nanoparticle residues form inside the shell. The diffusion of the melted Ag nanoparticles takes place at the same time, which results in the formation of Fe_2_O_3_ hollow spheres decorated with much smaller Ag nanoparticles.

**Figure 2:**
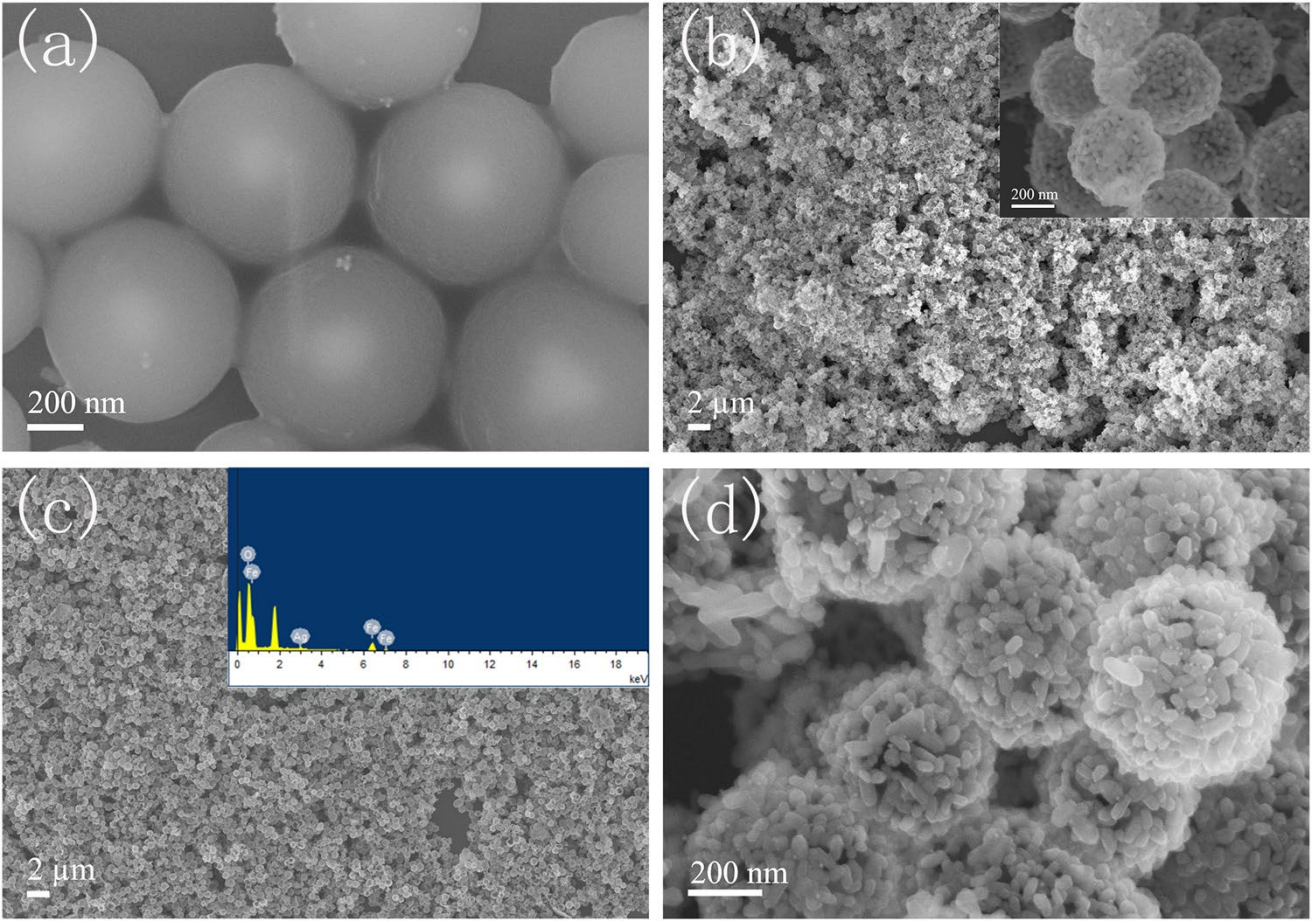
SEM images of the samples (**a**) Ag@C templates, (**b**) Fe_2_O_3_ nanospheres, (**c**) low magnification Ag-Fe_2_O_3_ composites and EDS spectrum of Ag-Fe_2_O_3_ composites, (**d**) high magnification Ag-Fe_2_O_3_ composites.

Figure 2(a) shows SEM images of the as-prepared Ag@C templates with good dispersibility and the diameter of Ag@C templates is about 650–700 nm. After the Fe_2_O_3_ growth and annealing procedure, Ag-Fe_2_O_3_ hollow spheres are obtained. As seen in Fig. 2(b,c and d), the as-synthesized Fe_2_O_3_ and Ag-Fe_2_O_3_ nanospheres are monodisperse and comparatively uniform in size. Figure 2(b) displays Fe_2_O_3_ nanospheres have a hollow structure. High magnification of Fe_2_O_3_ nanospheres inset in Fig. 2(b) reveals that the diameter of Fe_2_O_3_ hollow spheres is about 550 nm which is similar with Ag-Fe_2_O_3_. Low magnification of Ag-Fe_2_O_3_ shown in Fig. 2(c) indicates that the sample has more uniform morphology than Fe_2_O_3_. The EDS spectrum reveals that a small amount of Ag exists in the Fe_2_O_3_ nanospheres with the weight percentage of Ag about 3.9%. A small amount of carbon exists in Ag-Fe_2_O_3_ composites after annealing process, and the present of carbon is favorable to the electrochemical properties of the composite. The specific weight/atomic percentages for each element obtained from EDS measurement are given in Table S1 in supporting information (SI). High magnification of SEM illustrated in Fig. 2(d) demonstrates the diameter of Ag-Fe_2_O_3_ nanospheres is about 600 nm. It can also be obviously observed that Ag-Fe_2_O_3_ hollow nanospheres are composed of a number of Fe_2_O_3_ nanorods with approximately 60–70 nm long and 30–40 nm wide. Additionally, ultra-small Ag nanoparticles are evenly distributed on the surface of Fe_2_O_3_ spheres.

**Figure 3:**
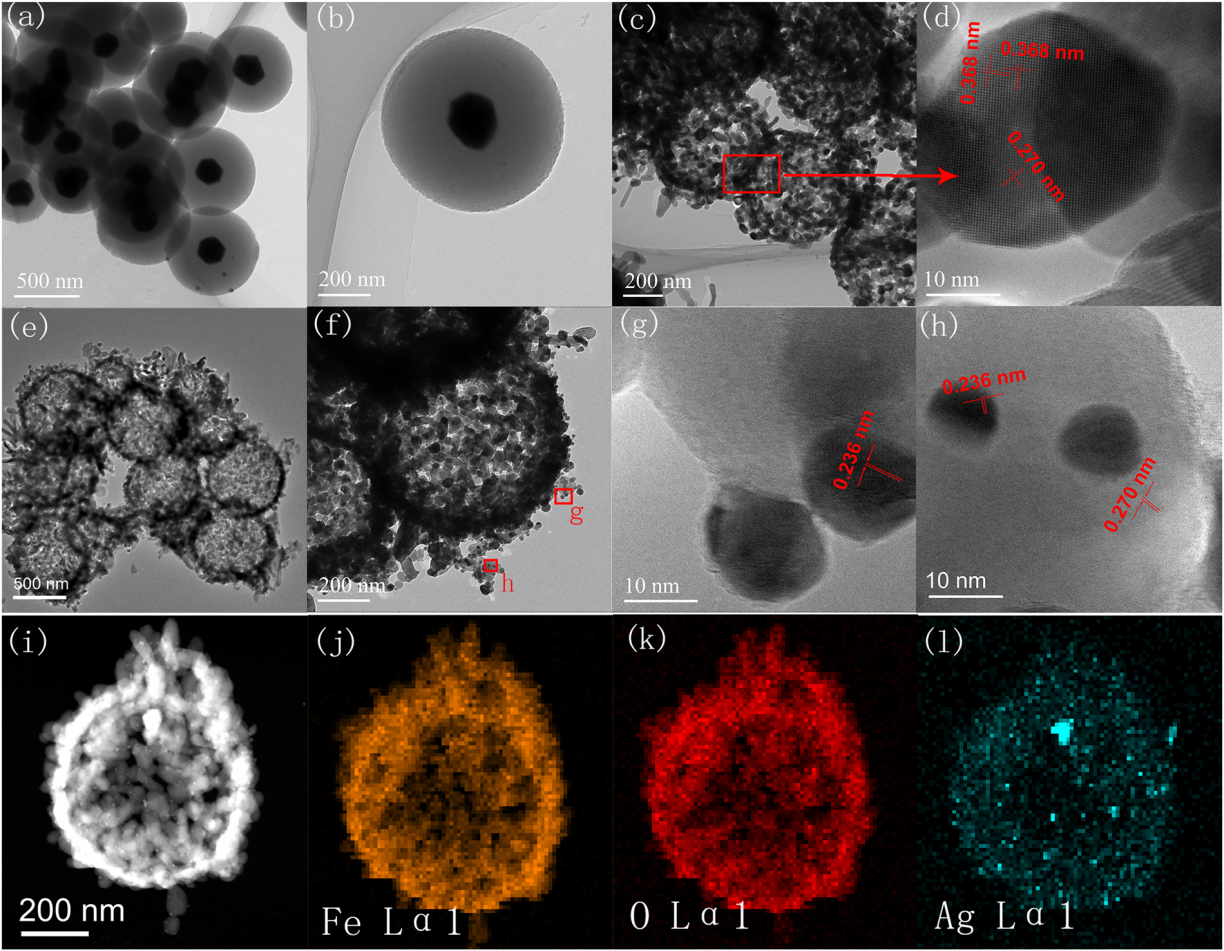
(**a**,**b**) TEM of Ag@C templates. (**c**) TEM of Fe_2_O_3_ nanospheres. (**d**) HRTEM of Fe_2_O_3_ nanospheres. (**e** and **f**) TEM of Ag-Fe_2_O_3_ composites. (**g** and **h**) HRTEM of Ag-Fe_2_O_3_ composites. (**i–l**) Phase mapping of Ag Lα1, Fe Lα1 and O Lα1.

The morphology and structure of the materials are further studied by TEM, and HRTEM measurements. TEM images in Fig. 3(a and b) show Ag@C templates are uniform, each particles consists of Ag core about 150 nm in diameter and carbon shell with thickness about 230 nm. Figure 3(c) shows the Fe_2_O_3_ hollow spheres formed by the accumulation of Fe_2_O_3_ nanorods. HRTEM observations are carried out to investigate the crystalline structure. The lattice spacing shown in Fig. 3(d) are calculated to be 0.368 nm and 0.270 nm which agree with the spacing between (012) and (104) planes of α-Fe_2_O_3_. Figure 3(e and f) reveal Ag-Fe_2_O_3_ composites have a hollow structure and the wall thickness is about 60 nm. The diameter of the Ag-Fe_2_O_3_ nanospheres is about 600 nm, in agreement with SEM result in Fig. 2(d). Ag nanoparticles distributed on the surface of Fe_2_O_3_ show a size about 10 nm, much smaller than that of Ag core in Ag@C templates. This result may be due to the disintegration and diffusion of Ag core during the annealing process. Figure 3(g and h) present HRTEM images with lattice fringes take from Ag-Fe_2_O_3_ spheres. Figure 3(g) reveals the interlayer spacing of 0.236 nm corresponded well to the spacing of Ag (111) planes. Moreover, Fig. 3(h) indicates the lattice spacing of 0.236 nm and 0.270 nm are respectively consistent with the spacing of Ag (111) and α-Fe_2_O_3_ (104) planes. Figure 3(i–l) show the phase mapping of Ag Lα1, Fe Lα1 and O Lα1 tested by EDS analyzer equipped in TEM instrument, which reveal a uniform distribution of Ag elements (green) in Fe_2_O_3_ hollow structure.

**Figure 4:**
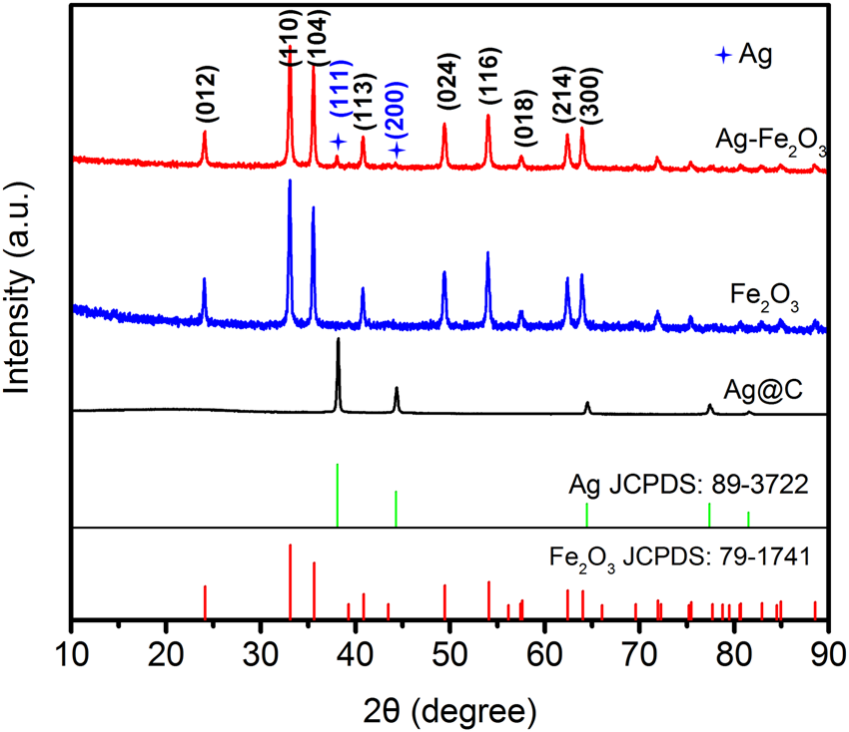
The XRD patterns of Ag@C templates, Fe_2_O_3_ nanospheres and Ag-Fe_2_O_3_ composites.

The crystalline nature of the as-prepared Ag@C template, Fe_2_O_3_ nanospheres and Ag-Fe_2_O_3_ composites are examined by X-ray diffraction (XRD). As shown in Fig. 4, it can be seen that the diffraction peaks of Ag@C template appearing at 38.1°, 44.3°, 64.4°, 77.4° and 81.6°, which are corresponding to the cubic structure of Ag (JPCDS No. 89–3722). For Ag-Fe_2_O_3_ composites, all the diffraction peaks are indexed to the hexagonal structure of α-Fe_2_O_3_ phase (JCPDS No. 79–1741). The position of the diffraction peaks indicates the type of Fe_2_O_3_ is hematite. For better learning the type of Fe_2_O_3_, we also carries out Raman test. The result in Fig. S1 shows the product we obtained is hematite^34–38^, in agreement with XRD results in Fig. 4. Moreover, two relatively weak peaks locate at 38.1° (111) and 44.3° (200) are discernible, which belong to the Ag nanoparticles. Fe_2_O_3_ nanospheres are used as a comparative sample, XRD patterns is similar to that of Ag-Fe_2_O_3_ composites except for the peaks of Ag. Peaks from other phases are not detected indicating high purity of the samples. The carbonaceous layer in the Ag@C templates is reducible during the high-temperature annealing process, which will protect Ag from oxidation during the diffusion process. Consequently, Ag nanoparticles are obtained in the final product rather than Ag_2_O^33^.

**Figure 5:**
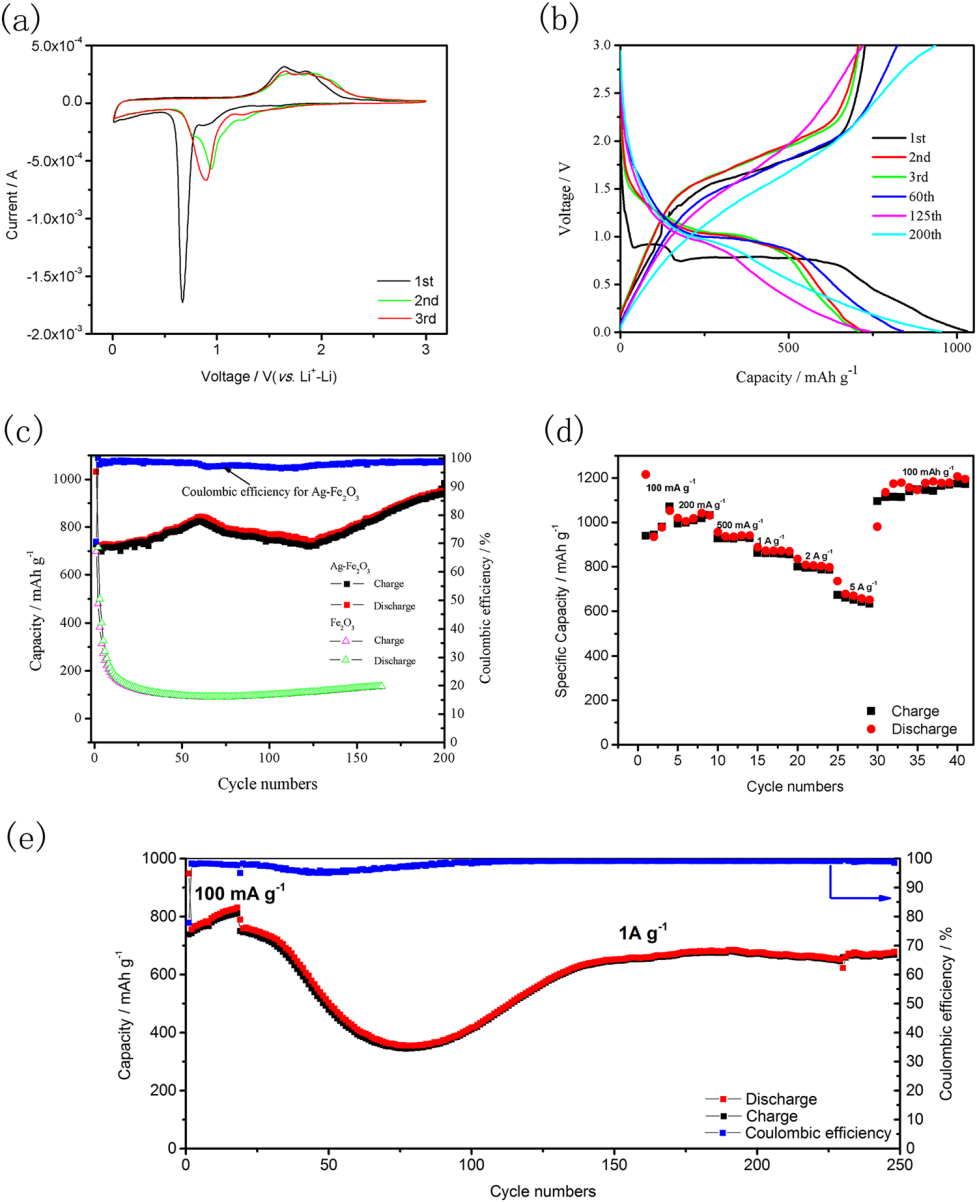
Electrochemical measurements (**a**) CV curves of Ag-Fe_2_O_3_ electrodes at a scan rate of 0.1 mV s^−1^ between 0.01 and 3.0 V, (**b**) Discharge and charge voltage profiles of Ag-Fe_2_O_3_ electrode at a current density of 100 mA g^−1^ for different cycles, (**c**) The cycling performance of Fe_2_O_3_ nanospheres and Ag-Fe_2_O_3_ composites electrode at 100 mA g^−1^, (**d**) Rate capabilities of Ag-Fe_2_O_3_ composites electrode, (**e**) The cycling performance of Ag-Fe_2_O_3_ composites electrode at 1 A g^−1^.

The electrochemical properties of Ag-Fe_2_O_3_ electrode for LIBs are systematically measured by using a lithium foil electrode as reference electrode in the coin-cell batteries. The cyclic voltammetry (CV) analysis of Ag-Fe_2_O_3_ electrode collect at a scan rate of 0.1 mV s^−1^ between 0.01 and 3.0 V is illustrated in Fig. 5(a). The CV curves of the sample are similar to the previously reported results^13, 39, 40^. Plenty of differences between the first cycle and consecutive cycles are noticed. At the first cycle, there are two peaks appear in the cathodic sweep. The strong reduction peak observed at 0.67 V corresponds to the reduction of Fe (III) to Fe (0) and the formation of solid-electrolyte interface (SEI), while a broad peak locate at 0.87 V can be ascribed to Li ions insertion into Fe_2_O_3_ without structural change as follows:

$${{\rm{Fe}}}_{2}{{\rm{O}}}_{3}+{{\rm{xLi}}}^{+}+{\rm{x}}{e}^{-}\to {{\rm{Li}}}_{{\rm{x}}}{{\rm{Fe}}}_{2}{{\rm{O}}}_{3}$$

During the anodic scan, two extended peaks appear at 1.64 V and 1.80 V, which is attributed to the oxidation of Fe (0) to Fe (II) and further oxidation to Fe (III)^39–41^. In subsequent cycles, the full lithiation potential is characterized by a higher voltage at 0.95 V. The change is mainly in virtue of the improved kinetics of Ag-Fe_2_O_3_ which can be resulted from inherent nanosize effects in the TMOs electrode during cycling. Worthy of nothing, the electrochemical behavior of the composites is largely sustained except the gradual changes in intensity or position of the peaks. These phenomena indicate the good cycling performance of Ag-Fe_2_O_3_ electrode. The CV curves of Fe_2_O_3_ nanospheres is shown in Fig. S2, which display similar electrochemical process with Ag-Fe_2_O_3_.

Figure 5(b) exhibits the discharge and charge voltage profiles of Ag-Fe_2_O_3_ electrode at a current density of 100 mA g^−1^ within a voltage window range of 0.01–3 V, which are in good agreement with the peaks of the CV curves in Fig. 5(a). A first overall discharge (lithiation) capacity is experimentally as large as 1030.9 mA h g^−1^, which is higher than the theoretical capacity (1007 mA h g^−1^). Two plateaus are observed during the first discharge. The peak located at 0.91 V corresponds to Li^+^ metal insertion into Ag-Fe_2_O_3_ electrode, and the followed long stage at 0.79 V indicates that the reduction of Fe (III) to Fe (0) and the formation of SEI. When the discharged electrode is recharged to 3.0 V, a smooth voltage plateau is observed at 1.3 V, then the voltage profile has a sudden rise from 2.1 V to 3.0 V, which finally reaches a charge capacity as high as 726.9 mA h g^−1^. The first initial coulombic efficiency is about 70.5%. This high initial irreversible loss may be due to the formation of SEI and structural change of electrode. Nevertheless, this initial coulombic efficiency of 70.5% is quite outstanding compared with some reports (no more than 50%)^42, 43^. In subsequent second and third discharge voltage profiles, the plateaus rise to 1.05 V. The 60th discharge curve shows a slope at 0.98 V and the capacity increases to 947.8 mA h g^−1^. Immediately after 60 cycles, a slight drop in specific capacity is recorded. The capacity decreases to 721.3 mA h g^−1^ at 125th cycle, which may be attributed to the crushing of the electrode. The plateau at 0.95 V of 125th cycle become shorter than previous cycles, which indicates a decreased capacity. Beyond 125th cycle, the capacity gradually increases to 953.2 mA h g^−1^ in the 200th cycle. The discharge curves’ decay slow down with aging below 0.95 V from 125th to 200th cycles, which may be ascribed to the increased capacity. Such a low voltage hysteresis owing to faster ion migration rate and a reformative energy efficiency is absolutely necessary for the commercialized development of TMOs-based electrodes^44, 45^.

Figure 5(c) shows the cycling performance of the Ag-Fe_2_O_3_ anode which is tested at a current density of 0.1 A g^−1^. For comparison, we also performs cycling experiment for the corresponding Fe_2_O_3_ anode. Worthy of nothing, the cycling process of Ag-Fe_2_O_3_ composites seems to undergo three different steps. Stage (A): from the 2nd cycle to 60th cycle, the anode of Ag-Fe_2_O_3_ exhibits a gradual enhancement of the lithium storage capacity. This increase in capacity may be due to the activation of the electrode and the formation of SEI during cycling. It takes several cycles to form stable SEI films on the discharge intermediates. These SEI films establish an intimate contact with connector which will improve the accessibility during the cycling. Stage (B): in the range of 60–125 cycle, the decomposition of electrode takes a predominant role^46, 47^. Therefore, a slight drop in specific capacity is recorded. Stage (C): beyond the 125th cycle, one can see the curve shows an obvious gradual increase of the capacity. An anomalous monotonic increase in the discharge capacity is observed from the 125th (721.3 mA h g^−1^) to 200th (953.2 mA h g^−1^) cycle. Indeed this phenomenon (increasing of capacity) has also been reported previously^48^. The high-rate lithium-induced reactivation often occur in the hollow structure metal oxide electrode. A recent research commanded by Hu *et al*. provides more direct evidence on the origin of additional capacity^49^. The increasing capacity during cycling is attributed to the reversible formation and decomposition of an organic polymeric gel-like film from kinetic activation in the electrode, which can coat the active materials and provide extra lithium interfacial storage sites to enhance the mechanical cohesion. The outside SEI layer may be broken, peeled off and reformed, resulting in the thick and unstable SEI layer during the cycling with a declining capacity. When the structure refinement, a thin and stable SEI film gradually forms without splinter, then, the re-activated electrode will exhibit an excellent cycling stability in a long cycle. In general, the decomposition of electrode caused by volume change during cycling leading to capacity loss, while the reversible formation and decomposition of an organic polymeric gel-like film will result in increased capacity. For the purpose of exploring the role of Ag nanoparticles in improving the cycling properties of materials, the cycling performance of Fe_2_O_3_ nanospheres is also measured at a current density of 0.1 A g^−1^. Fe_2_O_3_ anode delivers initial 1401 mA h g^−1^ discharge capacity and 697 mA h g^−1^ charge capacity with a coulombic efficiency of 49.8%, which is lower relative to the Ag-Fe_2_O_3_ composites. It shows about 80% coulombic efficiency at the first 5 cycles. After that the capacities of Fe_2_O_3_ maintain a stable value about 170 mA h g^−1^ during the following cycles (more than 150 cycles). Fe_2_O_3_ usually suffers from the problem of poor electronic conduction, which will cause serious polarization, resulting in instability during the cycling. In addition, particle pulverization caused by the great volume changes and the strong agglomeration during the charge and discharge process will all lead to a steep capacity fading^13, 40, 50^. As shown in Fig. S3(a and b), the sphere Fe_2_O_3_ structure is barely maintained after cycling, which also suggested large capacity fading. The result clearly indicates that the decoration of Ag nanoparticles significantly improves the electrochemical performance of the electrode. The ultra-small Ag nanoparticles evenly disperses on the surface of Fe_2_O_3_ nanospheres will protect the anode from pulverization during cycling, thereby enhancing the cycle stability of the electrode. In additional, the unusual cycle performance of the Ag-Fe_2_O_3_ composites electrode will also provide a case for future studies.

To further characterize the cycle performance of Ag-Fe_2_O_3_ anode, tests are performed at a current density of 0.1 A g^−1^ for 20 cycles, then increasing to 1 A g^−1^. As shown in Fig. 5(e), Ag-Fe_2_O_3_ anode exhibits capacity of 678 mA h g^−1^ after 250 cycles at 1 A g^−1^. Additionally, it is clear that the cycle at high current density is consistent with the trend at low current density except a slight difference in stage (B). This severe capacity decline trend may be ascribe to much serious electrode smash and irreversible reaction when the anode cycles at high current density. Metal oxides having a hollow structure can withstand a certain volume expansion at a low rate and during the first cycle. However, when cycling at a high-rate and longer cycles, the anode still subjected to severe mechanical degradation owe to the drastic volume changes inherently along with the conversion reaction.

A higher rate performance of LIBs electrode is particularly crucial, especially for high power density applications such as electric vehicles. Figure 5(d) shows the rate performance of the Ag-Fe_2_O_3_ hollow nanospheres at different current densities. Benefiting from the modification of Ag nanoparticles and hollow structures, the composites anode exhibits an excellent rate capability. When cycling at the current densities of 0.1, 0.2, 0.5,1, 2 and 5 A g^−1^, the electrode shows discharge capacities of 938.2, 1009.5, 925.7, 860.3, 794.0, and 650.8 mA h g^−1^, respectively. In addition, a high capacity of 1113 mA h g^−1^ can be achieved quickly when the current density change from 5 A g^−1^ to 0.1 A g^−1^. Such a remarkable result obtained from Ag-Fe_2_O_3_ electrode is better than most Fe_2_O_3_-based electrodes previously reported, involving Fe_2_O_3_/CNT, Fe_2_O_3_/GF and Fe_2_O_3_-carbon composites^51–54^. For comparison, the rate performance of Fe_2_O_3_ nanospheres is showed in Fig. S2(b). The specific capacity decline seriously with increasing current densities, suggesting that Ag nanoparticle incorporation and hollow structure have a significant effect on improving rate performance.

**Figure 6:**
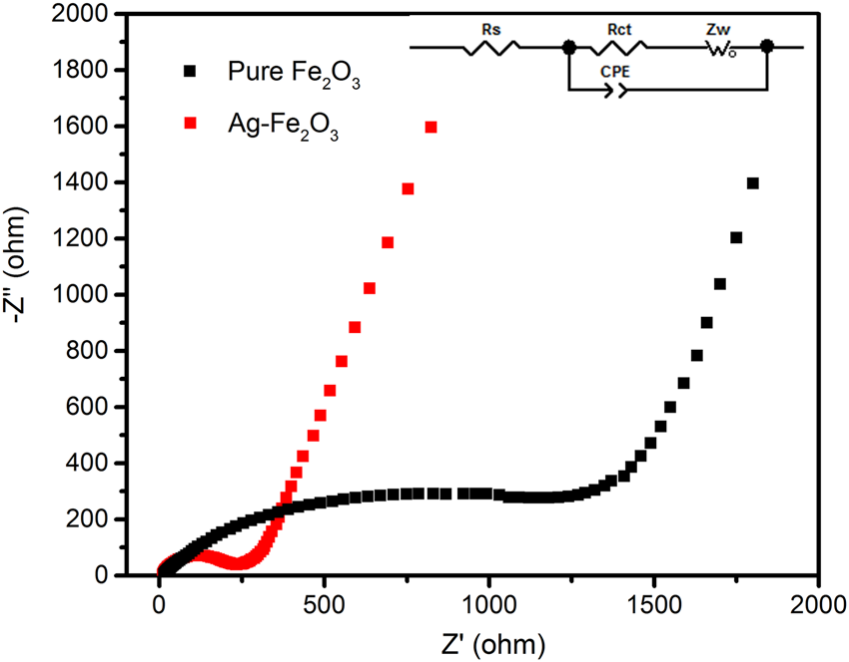
Nyquist plots of Fe_2_O_3_ and Ag-Fe_2_O_3_ composites electrode.

The electrochemical impedance spectra (EIS) of Fe_2_O_3_ and Ag-Fe_2_O_3_ electrode is conducted to demonstrate that the decoration of Ag nanoparticles can obviously improve the charge transfer kinetics, which are given in Fig. 6. The Nyquist plot consists of a semicircle at middle frequency and a sloped line at low frequency. The semicircle at middle frequency is associated with charge transfer resistance (*R*ct). Ohmic resistance (*R*s) is related to the contact resistance between the active material and current collector. The sloped line at low frequency range is influenced by ion diffusion (*Z*w). The Constant Phase Element (CPE) is the physical quantity used to describe the deviation of the parameters of capacitor C, which depending on the nature of the system being investigated. The Nyquist plots for Ag-Fe_2_O_3_ composites possesses much smaller diameters of the semicircles than that of the pure Fe_2_O_3_. Based on equivalent circuit, the *R*ct values of Ag-Fe_2_O_3_ and Fe_2_O_3_ are 230 Ω and 1500 Ω, respectively. The difference in *R*ct indicates that the decoration of Ag nanoparticles can significantly enhance the conductivity of the material and thus promotes the charge transfer kinetics.

**Figure 7:**
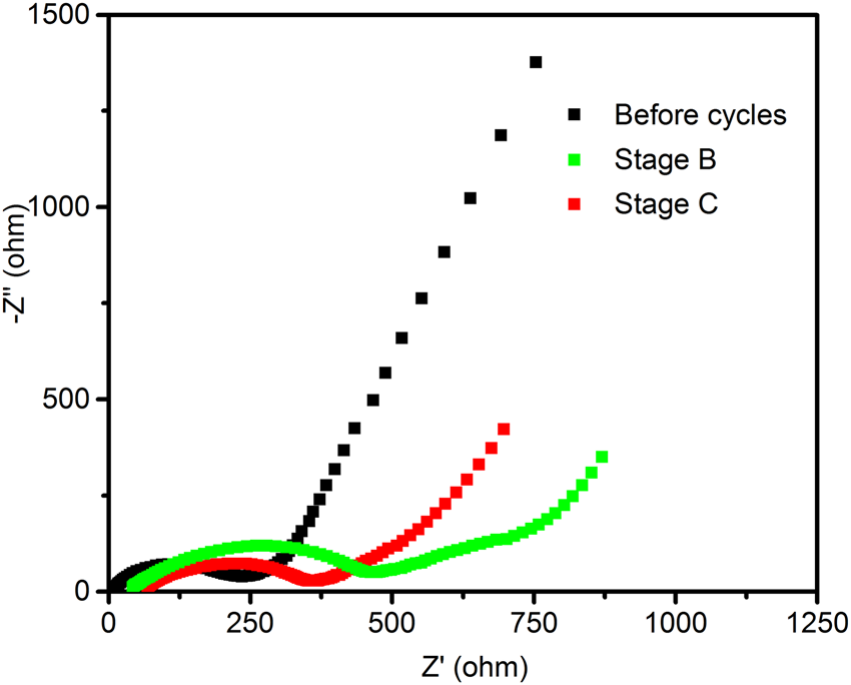
Nyquist plots of Ag-Fe_2_O_3_ composites electrode during stage (B) and stage (C) cycles.

Figure 7 exhibits the impedance analysis of Ag-Fe_2_O_3_ composites electrode in stage (B) and stage (C) cycles. The charge transport resistance characterized by the semicircle at medium frequencies of stage (B) (400 Ω) is higher than that in stage (C) (300 Ω), which is consistent with the cycle performance. Therefore, the capacity rise can be ascribed to the reversible formation of polymeric/gel-like layer and/or interfacial lithium storage. The EIS result is agree with the cycling process of Ag-Fe_2_O_3_ composites electrode is shown in Fig. 5(c).

We also carry out SEM characterization of Fe_2_O_3_ and Ag-Fe_2_O_3_ composites electrode after 200 cycles (Fig. S3). Spherical Fe_2_O_3_ is barely found after cycling, and they become aggregated as shown in Fig. S3(a and b). On the contrary, the spherical structure of Ag-Fe_2_O_3_ composites is almost maintained after cycling as shown in Fig. S3(c), and even a number of hollow spheres exist as shown in Fig. S3(d). Therefore, uniform distribution of Ag nanoparticles on the surface of Fe_2_O_3_ nanospheres will benefit to the cycle stability of Ag-Fe_2_O_3_ nanocomposite electrode.

## Conclusion

In summary, a hollow structure of Ag-Fe_2_O_3_ composites was synthesized by using Ag@C core-shell matrix as sacrificial templates and subsequent calcining process. In virtue of its hollow structure and the decoration of Ag nanoparticles over Fe_2_O_3_ nanospheres, the composites exhibited an improved cycling performance (~ 953.2 mA h g^−1^ at 100 mA g^−1^ after 200 cycles and 678 mA h g^−1^ at 1 A g^−1^ after 250 cycles). The Ag-Fe_2_O_3_ electrode also exhibited an extraordinary high-rate performance (~ 650.8 mA h g^−1^ at 5 A g^−1^). Hence, this work showed that exploring of Fe_2_O_3_ composites might open venues for the practical applications of TMOs anodes in the next-generation of high-performance Li-ion batteries.

## Methods

### Preparation of Ag@C and Carbon spheres templates

All the reagents in the experiment were analytical grade and used without further purification.

Ag@C templates were synthesized by a modified hydrothermal method^55^. 30 mL of 10 mM AgNO_3_ aqueous solution was added into 20 mL of 1 M glucose solution. After continual stirring for 30 min, the mixture was transferred to a 100 mL Teflon-lined autoclave and maintained at 180 °C for 6 h. After the autoclave naturally cooled down to the room temperature, the products were washed by water and ethanol respectively till the upper liquid become clear after centrifugation. Finally, the resulting products were oven-dried at 80 °C for 10 h. Furthermore, we also prepared carbon spheres in the same manner without addition of AgNO_3_.

### Preparation of Ag-Fe_2_O_3_ composites

0.08 g of as-prepared Ag@C templates were dispersed in 50 mL deionized water. After ultrasonication for 30 min, 0.278 g of FeSO_4_·7H_2_O and 0.3 g of sodium acetate (NaAc) were added into the solution. Subsequently, the solution was stirred vigorously for 24 h at room temperature. Then, the resultant composites were purified by repeated centrifugation and dispersion cycle, and finally dried at 80 °C for 10 h. The final products of Ag-Fe_2_O_3_ composites were placed into the muffle furnace and calcined at 600 °C for 2 h in air. For comparison, we also synthesized Fe_2_O_3_ nanospheres using the as-prepared carbon templates with a similar procedure.

### Materials characterization

Morphology and chemical compositions of the samples were characterized using scanning electron microscope (SEM, Zeiss SUPRA 55) equipped with an energy dispersive X-ray Spectroscopy (EDS, Oxford), transmission electron microscopy (TEM, JEOL JEM 2100) and high resolution transmission electron microscopy (HRTEM, JEOL JEM 2100), X-ray diffraction (XRD, Rigaku Ultima IV) were recorded on a Panalytical X-pert diffracto meter with Cu Kα irradiation.

### Electrochemical measurements

The electrochemical measurements were characterized using CR2025-type coin cells. Pure lithium foils were used as the counter and reference electrodes. The active materials were mixed with carboxyl methyl cellulose and carbon black in a weight ratio of 80:10:10. The mixture was pressed onto copper foil and dried under vacuum at 100 °C for 10 h. The active material loading of the electrodes was about 0.8–1 mg·cm^−2^. The coin-cell was assembled in an argon-filled glove box with oxygen contents less than 0.5 ppm. The electrolyte was 1 M LiPF_6_ in a mixture of EC, EMC, DMC (1:1:1, in v:v:v). A Celgard 2400 microporous polypropylene membrane was used as a separator. The cyclic voltammogram (CV) was performed by using a CHI 660E electrochemical workstation (Shanghai Chenhua Instrument Co., China) in the potential range of 0.01–3.0 V *vs*. Li/Li^+^ at a scan rate of 0.1 mV s^−1^. The electrochemical impedance spectroscopy (EIS) technique was measured at open circuit potential in a frequency range from 10^–2^ Hz to 10^5^ Hz.

### Data availability

All datasets generated or analysed during this study are included in this published article and its Supplementary Information files.

## Electronic supplementary material

Supporting Information
