# Supplementary material for: Templating synthesis of Fe2O3 hollow spheres modified with Ag nanoparticles as superior anode for lithium ion batteries
Source: Sci Rep. 2017 Aug 29;7:9657. doi: 10.1038/s41598-017-08773-6 (PMC5574980; doi:10.1038/s41598-017-08773-6)
Supplement: Supplementary file 1 — Supporting Information [file 41598_2017_8773_MOESM1_ESM.pdf]

Supporting Information

Templating synthesis of Fe_2O_3 hollow spheres modified with Ag nanoparticles as superior anode for lithium ion batteries

Xiaoping Lin[1], Jianmin Zhang[1], Xiaobin Tong[1], Han Li[1], Xi Pan[1], Peigong Ning[1], Qiuhong Li[1], *

[1]Pen-Tung Sah Institute of Micro-Nano Science and Technology, Xiamen University, Xiamen 361005, China. E-mail: liqiuhong@xmu.edu.cn; Fax: +86-0592-2187196; Tel: +86-0592-2187198

Table S1 Specific weight/atomic percentages for each element in Ag-Fe_2O_3 composites

Element	Weight Percentage/ %	Atomic percentage/%
C K	6.22	12.48
O K	44.51	67.07
Fe K	45.35	19.58
Ag L	3.92	0.88
Total	100.00	

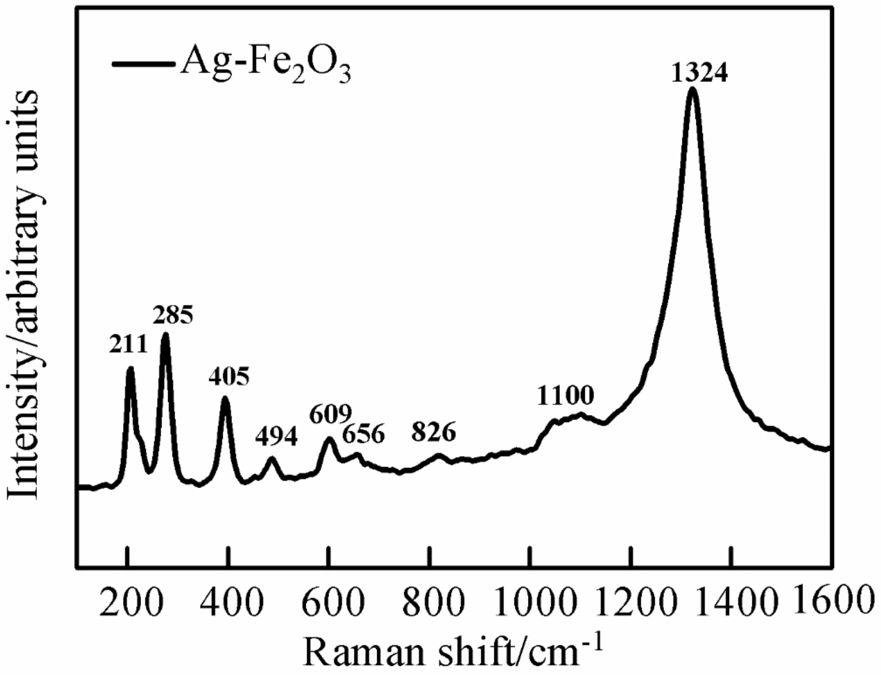

Fig. S1 Raman spectra of Ag- Fe_2O_3 composites

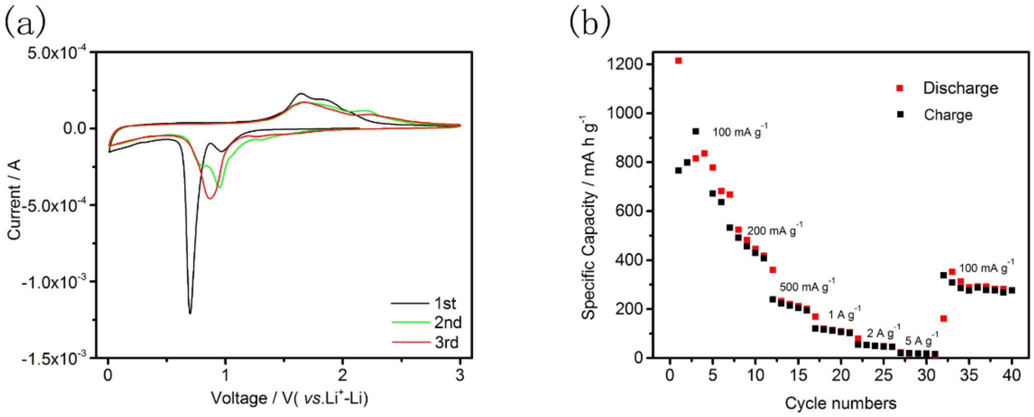

Fig. S2 Electrochemical measurements of pure Fe_2O_3 (**a**) CV curves of pure Fe_2O_3 electrodes at a scan rate of 0.1 mV s^{-1} between 0.01 and 3.0 V, (**b**) Rate capabilities of Fe_2O_3 electrode.

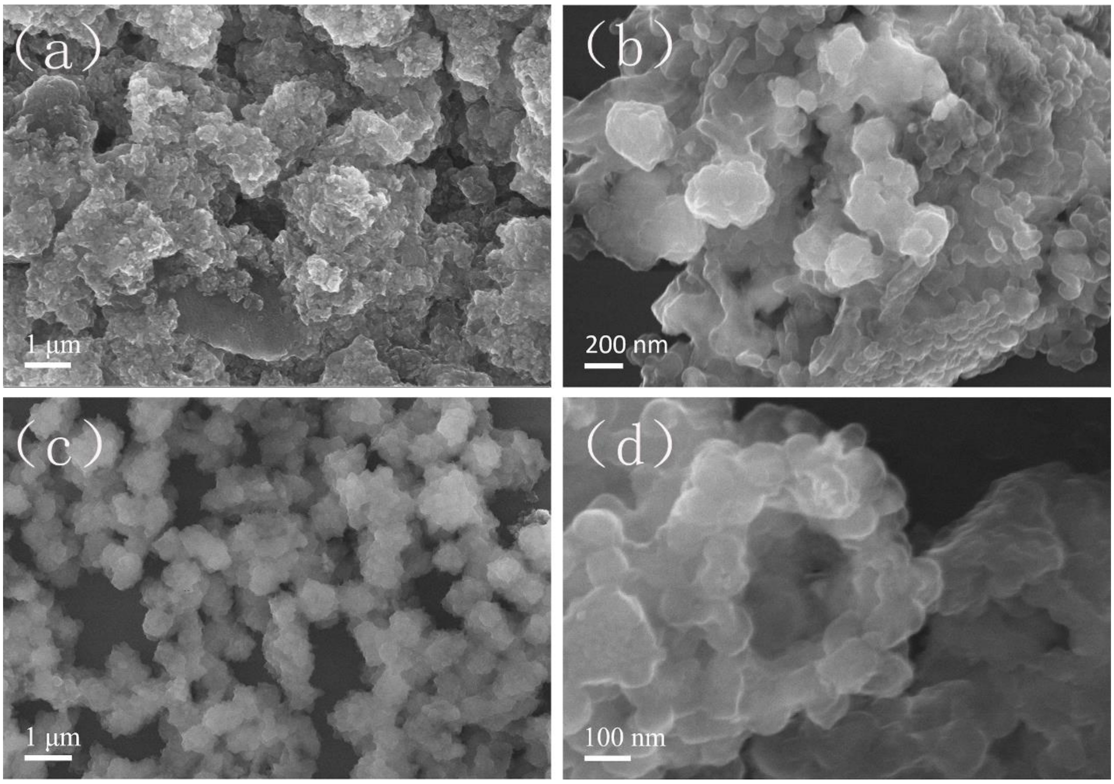

Fig. S3 SEM image of samples after 200 cycles. **(a) (b)** Fe_2O_3 nanospheres, **(c) (d)** Ag-Fe_2O_3 composites.
